# Divergent Dynamics and Functions of ERK MAP Kinase Signaling in Development, Homeostasis and Cancer: Lessons from Fluorescent Bioimaging

**DOI:** 10.3390/cancers11040513

**Published:** 2019-04-10

**Authors:** Yu Muta, Michiyuki Matsuda, Masamichi Imajo

**Affiliations:** 1 Department of Pathology and Biology of Diseases, Graduate School of Medicine, Kyoto University, Kyoto 606-8051, Japan; yumuta@kuhp.kyoto-u.ac.jp (Y.M.); matsuda.michiyuki.2c@kyoto-u.ac.jp (M.M.); 2 Department of Gastroenterology and Hepatology, Graduate School of Medicine, Kyoto University, Kyoto 606-8507, Japan; 3 Laboratory of Bioimaging and Cell Signaling, Graduate School of Biostudies, Kyoto University, Kyoto 606-8501, Japan; 4 Institute for Chemical Reaction Design and Discovery (WPI-ICReDD), Hokkaido University, Hokkaido 001-0021, Japan

**Keywords:** ERK MAP kinase, molecular activity dynamics, bioimaging, cancer

## Abstract

The extracellular signal-regulated kinase (ERK) signaling pathway regulates a variety of biological processes including cell proliferation, survival, and differentiation. Since ERK activation promotes proliferation of many types of cells, its deregulated/constitutive activation is among general mechanisms for cancer. Recent advances in bioimaging techniques have enabled to visualize ERK activity in real-time at the single-cell level. Emerging evidence from such approaches suggests unexpectedly complex spatiotemporal dynamics of ERK activity in living cells and animals and their crucial roles in determining cellular responses. In this review, we discuss how ERK activity dynamics are regulated and how they affect biological processes including cell fate decisions, cell migration, embryonic development, tissue homeostasis, and tumorigenesis.

## 1. Introduction

**Figure 1:**
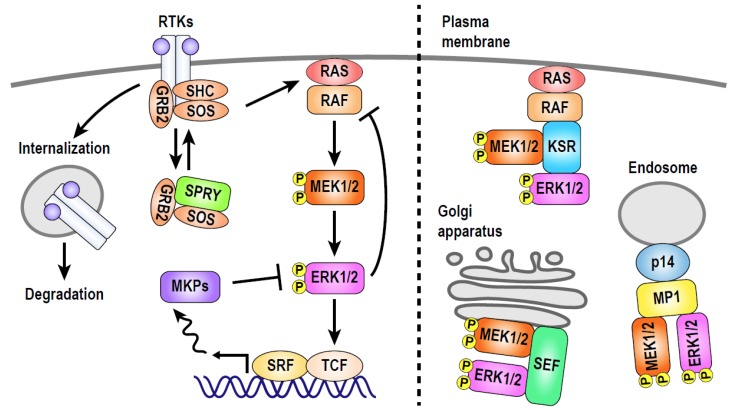
Factors affecting spatiotemporal dynamics of extracellular signal-regulated kinase (ERK) activity. The temporal patterns of ERK activation are defined by several feedback mechanisms. Ligand-induced activation of receptor tyrosine kinases (RTKs) causes their internalization and the following degradation, shutting off the input signals. Upon RTK activation, Sprouty (SPRY) family proteins are phosphorylated and then bind to growth factor receptor-bound protein 2 (GRB2) and son of sevenless (SOS), which inhibits the recruitment of these proteins to plasma membrane to limit the duration of ERK activation. When ERK is activated, it phosphorylates and suppresses functions of its upstream activators, including RAF and SOS. Moreover, transcriptional induction of MAP kinase phosphatases (MKPs) by the ternary complex factor (TCF)-serum response factor (SRF) complex serves as a mechanism for more delayed feedback inhibition of ERK signaling. In addition to the temporal regulation, distribution of activated ERK can be also controlled by several molecules that tether ERK to specific subcellular locations. For instance, KSR, MP1, and SEF tether both MEK and ERK to the plasma membrane, endosome, and Golgi apparatus, respectively.

The extracellular signal-regulated kinase (ERK) pathway is an evolutionarily conserved signaling pathway that regulates a variety of cellular processes including cell survival, proliferation, and differentiation [1,2,3,4]. The core of this pathway is a kinase cascade consisting of three classes of kinases, RAF, MEK and ERK. Upon activation of many receptor tyrosine kinases (RTKs), RAF is activated through the binding to the GTP-bound form of RAS, and phosphorylates MEK. The phosphorylated/activated MEK, then, phosphorylates both threonine and tyrosine residues of ERK in its activation loop. Once activated, ERK could phosphorylate hundreds of cytoplasmic and nuclear proteins, including transcription factors and cytoskeletal proteins, reflecting its diverse roles in various biological processes. Thus, in order to fulfill specific function, ERK activity is strictly regulated by many mechanisms depending on the cellular context (Figure 1). For instance, the ERK pathway is regulated by multiple feedback loops at different levels of the signaling cascade [5,6], which defines temporal dynamics of ERK activity. The duration of ERK activity is also controlled by several mechanisms, including internalization and degradation of RTKs, inactivation by specific phosphatases, and inhibition of adaptor protein recruitment by Sprouty (SPRY) family proteins (Figure 1) [7,8,9]. In addition to the temporal regulation, compartmentalization of the active ERK protein through the binding to scaffold and/or adaptor proteins (e.g., KSR, MP1, SEF, etc.) provides another level of complexity to the regulation of ERK signaling, enabling selective phosphorylation of a restricted set of ERK substrates at specific subcellular locations (Figure 1) [10,11,12,13,14,15]. The expression levels and function of the ERK signaling regulators are often modulated by transcription factors and other signaling pathways in a context-dependent manner [16,17]. Thus, spatial and temporal dynamics of ERK activity are tightly controlled by multiple mechanisms to evoke specific patterns of its substrate phosphorylation, which has been assumed to underlie divergent cellular outputs upon ERK pathway activation. Until recently, however, spatiotemporal dynamics of the ERK pathway in living animals and their physiological significance remained largely unknown due to technical difficulties in detecting ERK activity in vivo.

Excessive or inappropriate activation of the RTK/RAS/ERK pathway has been implicated in the initiation and progression of human cancers. Constitutive activation of this pathway is frequently observed in many kinds of cancers, and has been shown to confer a proliferative advantage and the malignant phenotypes on cancer cells [18,19,20,21,22]. Therefore, pharmacological targeting of the RTK/RAS/ERK pathway has been considered a promising strategy for cancer therapy [23]. So far, several monoclonal antibodies and kinase inhibitors have already been used in the clinic for the treatment of various cancers [23,24]. However, in spite of the apparent significance of the ERK pathway in many cancers, it remains elusive how ERK activity dynamics are altered at the single cell level and how it affects cellular functions during oncogenesis. 

Intensive study over the past few decades has uncovered that two different modes of ERK activation, transient or sustained, are important determinants of cellular responses [25]. However, most of these studies depended on classical biochemical analyses, which told only total molecular activity in a bulk population of cells at a certain moment. Thus, ERK activity dynamics in each cell had long been elusive. Recent advances in fluorescent bioimaging have opened new avenues for analyzing activity of various signaling molecules in real-time at the single-cell level [26,27,28]. Emerging evidence from bioimaging studies suggests that individual cells exhibit complex spatial and temporal ERK activity dynamics not only in cultured cells in vitro but also in live animals in vivo [29,30,31,32,33,34]. Furthermore, these studies have also shown that ERK activity dynamics play a key role in embryonic development and tissue homeostasis in adult life through regulation of many cellular activities, such as proliferation and migration. Recently, we have also uncovered complex ERK activity dynamics in the intestinal epithelium of living mice and found that alterations in ERK activity dynamics underlie specific traits of intestinal tumor cells [35]. Here, we provide an update on our current understanding of biological significance and regulatory mechanisms of ERK activity dynamics. We will first review a classical view of the ERK activity duration from biochemical analyses of a bulk cell population, and highlight how recent single-cell analyses with bioimaging techniques advance our understanding of ERK activity dynamics. We will then focus on physiological significance of ERK activity dynamics in several biological contexts, especially focusing on intestinal tumorigenesis.

## 2. A Classical View of the Duration of ERK Signaling: Sustained or Transient Activation

The duration of ERK activation, as well as its magnitude, has long been considered an important determinant of cellular responses [36,37]. A well-known example supporting this paradigm is growth factor-dependent cell fate decisions of rat pheochromocytoma (PC12) cells [38]. In PC12 cells, stimulation with different growth factors triggers distinct cellular responses. Treatment with nerve growth factor (NGF) induces neural differentiation, while stimulation with epidermal growth factor (EGF) promotes cell proliferation [38,39]. The different responses of PC12 cells have been attributed to distinct temporal patterns of ERK activation: NGF causes sustained ERK activation lasting several hours, whereas EGF triggers only transient and short-lived activation [40,41,42]. The difference in ERK activity dynamics can be accounted for in part by the amount and dynamics of receptors for these growth factors present at the cell surface. Upon activation by ligands, EGF receptor (EGFR) is rapidly internalized and degraded, whereas TrkA, a receptor for NGF, is translocated to long-lived signaling endosomes, where it avoids degradation and mediates sustained signaling [8,43]. When EGFR is overexpressed, EGF stimulation drives sustained but not transient ERK activation and induces differentiation of PC12 cells [44], suggesting that the decrease in the cell surface EGFR upon ligand stimulation is a critical limiting factor for the duration of ERK activation. In addition, different small G proteins mediate ERK activation downstream of these receptors. Although both EGF and NGF cause transient activation of Ras, only NGF induces sustained activation of Rap1, which drives sustained ERK activation [45,46]. Thus, in PC12 cells, differences in dynamics of the upstream receptors and small G proteins involved determine the duration of ERK activation upon ligand stimulation. 

Then, how is the duration of ERK activation linked to specific cellular responses? An answer to this question is hierarchical control of transcription factors by ERK activity. For instance, ERK activation rapidly induces expression of an immediate-early gene (IEG), c-fos, through activation of the ternary complex factors (TCFs)-serum response factor (SRF) complex [47]. The newly synthesized c-fos protein is unstable at first, but stabilized by ERK-mediated phosphorylation [48]. Thus, only sustained ERK activation induces phosphorylation and accumulation of c-fos protein, which in turn triggers the following transcriptional program required for neuronal differentiation of PC12 cells. Similarly, stability of several other IEGs is also regulated by ERK-mediated phosphorylation [48,49].

The temporal dynamics of ERK activity have also been implicated in the control of cellular responses in many cell types [36,37,50]. In fibroblasts, sustained, but not transient, activation of ERK induces cell proliferation. In this context, sustained ERK activation is required for continuous suppression of antiproliferative genes, as well as induction of proliferation-promoting genes [51]. In T cell development, duration of ERK activity regulates the CD4 versus CD8 lineage commitment [50]. The duration of ERK activation also participates in embryonic patterning in *Xenopus laevis*, where sustained ERK activation induces dorsal mesodermal genes by controlling accumulation of fos protein [17]. Sprouty2, a temporal regulator of the ERK pathway, has been shown to limit the duration of ERK activation to establish dorsoventral patterning in Xenopus embryos [17]. As highlighted by a vast array of previous studies, biological significance and regulatory mechanisms of two classical modes of ERK activation, sustained or transient, have long attracted considerable attention as one of the key factors in cell fate decisions.

## 3. Complex Single-Cell Dynamics of ERK Activity in Live Cells and Animals

The observations that temporally distinct patterns of ERK activation lead to different cellular outputs have motivated development of biosensors that can detect subtle changes in ERK activity in real time at the single-cell level. Thus, great efforts have been made to develop various kinds of ERK activity reporters based on different mechanisms of action, leading to development of two classes of highly sensitive biosensors for ERK activity. The first one is Förster/fluorescence resonance energy transfer (FRET)-based biosensors [52,53]. FRET is defined as a process in which the excitation energy absorbed by one fluorophore (donor) is transferred to another fluorophore (acceptor) without radiation, resulting in fluorescence emission from the acceptor molecule. The key feature of FRET is that it occurs only when the two fluorophores are in very close proximity, which makes it useful in detecting molecular interaction in living cells. In general, the FRET biosensors for ERK activity contain two fluorescent proteins, usually CFP and YFP variants, the substrate sequence of ERK, and a phospho-serine/threonine-binding domain. When ERK is activated, it phosphorylates the substrate sequence in the biosensor. This phosphorylation induces intramolecular interaction between the phosphorylated substrate and the phospho-serine/ threonine-binding domain, which brings CFP and YFP into close proximity and allows FRET between these fluorescent proteins. Thus, ERK activity can be evaluated quantitatively by measuring FRET efficiency, which is often represented as the ratio of FRET and CFP fluorescence in actual measurements. The other type of biosensors named kinase translocation reporters (KTRs) contains the substrate sequence of ERK and both nuclear localization signal (NLS) and nuclear export signal (NES). Phosphorylation of the substrate sequence suppresses the activity of NLS and enhances that of NES, leading to translocation from the nucleus to cytoplasm [54]. Thus, kinase activity can be measured in the form of nucleocytoplasmic shuttling of the biosensors. The single-color nature of KTR biosensors enables multiplexed imaging of different kinases, but cannot, in principle, measure kinase activity at the specific subcellular compartment. In this respect, the FRET-based ERK biosensors targeted to specific subcellular compartments by localization signals enable to elucidate spatial dynamics of ERK activity in living cells.

Live cell imaging with ERK biosensors has uncovered that, even under the conventional culture conditions, cells exhibit temporally complicated patterns of ERK activity characterized by spontaneous and stochastic pulsatile ERK activation and its propagation to adjacent cells [29,30]. Several studies have suggested the involvement of RAF and EGFR in the generation of stochastic ERK activity pulses [29,32]. The propagation of ERK activity pulses is blocked by inhibitors of matrix metalloproteinases and EGFR [29], suggesting that shedding of EGFR ligands mediates the propagation. Notably, in addition to amplitude, frequency of ERK activity pulses has been shown to be a key determinant of cell proliferation [29,30]. Reconstruction of ERK activity dynamics by optogenetic approaches has shown that the repetitive, pulsatile ERK activation leads to the efficient induction of certain IEGs [29,55], suggesting that temporal differences in ERK activity dynamics could be decoded by the downstream transcription factors including the TCF/SRF complex. In addition to the control of cell proliferation, ERK activity dynamics also play a critical role in regulating cell migration. In classical wound-healing assay using cultured epithelial cells, two distinct types of waves of pulsatile ERK activity occur from the wound edge and in regions distant from the wound [33,56]. Both waves orient collective cell migration in a direction opposite to the waves [33]. Thus, complex ERK activity dynamics shaped by pulsatile ERK activation and its propagation regulate important cellular processes, such as proliferation and migration, in cultured cells. 

Live imaging with ERK biosensors has also been applied to in vivo studies of ERK activity, leading to elucidation of complex ERK activity dynamics and their physiological significance in diverse biological processes. Notably, ERK activity pulses and their lateral propagation were not specific to particular in vitro culture conditions, but also observed in living animals in vivo. For instance, in vivo imaging of transgenic mice expressing the FRET biosensor for ERK [57] has uncovered that spontaneous, pulsatile ERK activation and its wave-like propagation occur in several mammalian tissues. In the epidermis, ERK activity occasionally propagates in a radial fashion across cells and its frequency is associated with cell cycle progression from G2 to M phase [31]. Thus, the propagation of ERK activity might represent a novel mechanism to synchronize cell proliferation in the tissue. When injured, ERK activity is propagated as trigger waves from the wound edge in an opposite direction to cell migration [31], as in the case of the wound-healing assay using a cultured epithelial cell sheet. The ERK activity dynamics also regulate migration of immune cells. In the inflamed intestine, ERK activity is rapidly elevated in neutrophils to promote their submucosal infiltration and migration, when they adhere and spread on the endothelial cells [58]. By contrast, in the mouse thymus, ERK activity negatively regulates motility of thymocytes, suggesting that ERK activity could differentially regulate cellular motility depending on the cell types [59]. In addition to the mouse tissues, complex ERK activity dynamics has also been implicated in the control of embryonic development, tissue homeostasis and cell fate decisions in other model organisms, including the nematode *Caenorhabditis elegans*, fruit fly *Drosophila melanogaster*, and zebrafish *Danio rerio* [34,60,61,62]. Altogether, these findings highlight significance of ERK activity dynamics in the control of cellular behaviors and intercellular communication essential for collective cellular responses across species and organs.

## 4. ERK Activity Dynamics in Tumorigenesis

Oncogenic activation of the RTK/RAS/ERK pathway, which is often mediated by mutations in RAS, RAF or the upstream RTK genes, underlies a large proportion of human tumors [18,20,21,22]. These mutations have been shown to drive aberrant activation of ERK, resulting in inappropriate proliferation of tumor cells. In addition to this, these mutations could also alter the kinetics of ERK signaling activation in tumor cells [63]. For instance, oncogenic B-RAF mutations decelerate both the activation and inactivation rates of ERK activity, resulting in slow ERK pathway kinetics [63]. The changes in ERK activity kinetics allow cancer cells with these mutations to proliferate in response to transient upstream inputs, which are not sufficient to induce proliferation of normal cells: in these cancer cells, ERK activity generated by transient inputs could be prolonged enough to induce cell proliferation. Although these observations have been made in cell culture experiments, it would be plausible that changes in dynamics, as well as those in magnitude, of ERK activity play some roles in tumorigenesis. 

**Figure 2:**
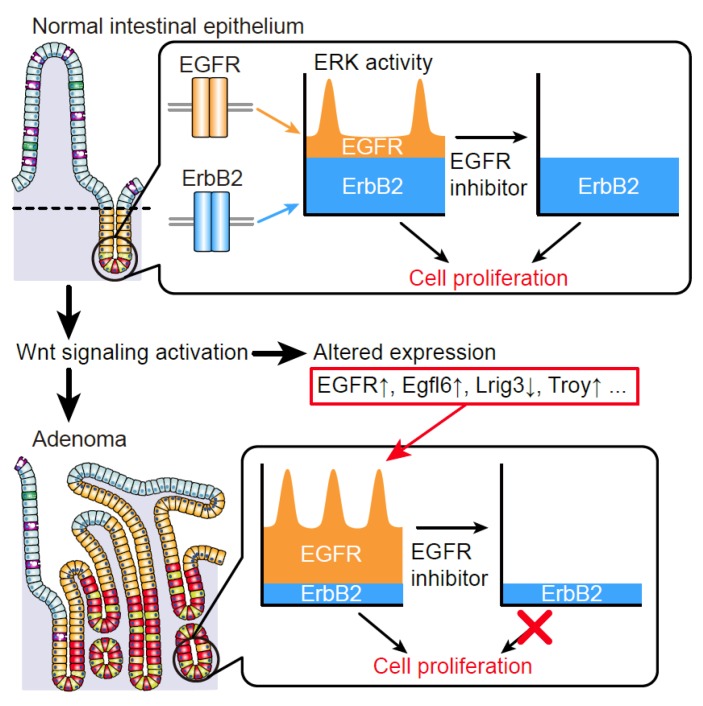
ERK activity dynamics in intestinal homeostasis and tumorigenesis. In the normal intestinal epithelium, ERK signaling dynamics are defined by EGFR-dependent pulsatile activity and ErbB2-dependent basal activity. During intestinal tumorigenesis, deregulated activation of Wnt signaling induces alterations in the expression levels of EGFR and its regulators, thereby enhancing EGFR signaling. The enhancement of EGFR signaling increases frequency of ERK activity pulses and promotes cell proliferation. At the same time, contribution of EGFR signaling to basal ERK activity is increased in tumor cells, which renders cells highly dependent on EGFR signaling. Thus, in the intestinal epithelium, tumor cells are more susceptible for pharmacological inhibition of EGFR signaling than normal cells.

In support of the importance of ERK activity dynamics in tumorigenesis, we have recently uncovered that changes in ERK activity dynamics underlie tumor-specific traits in the intestinal epithelium [35]. The significance of the ERK pathway in the intestinal epithelium has been well documented both in homeostasis and tumorigenesis. For instance, mice lacking EGFR or several ligands for EGFR show retarded growth throughout the intestine [64,65], while dysregulated activation of EGFR caused by depletion of Lrig1, a negative regulator of EGFR, leads to intestinal stem cell expansion and tumor formation [66,67]. Moreover, excessive activation of EGFR-ERK signaling has been implicated in human colorectal cancers (CRCs) and regimens containing anti-EGFR antibodies have been suggested as initial therapy in non-operable CRCs without KRAS mutation [21,22]. Although these studies have shown a critical role of the ERK pathway in intestinal homeostasis and tumorigenesis, ERK activity dynamics in normal and tumor intestinal epithelial cells remained elusive. By using transgenic mice expressing the FRET biosensor for ERK [57], we recently succeeded in visualizing ERK activity in the intestinal epithelium of living mice [35]. Intestinal epithelial cells (IECs) at the crypts exhibit sporadic ERK activity pulses, which are generated by spontaneous firing in each cell or propagation from adjacent cells. In addition to the pulsatile activity, IECs also exhibit the sustained, basal activity. Similar ERK activity dynamics could be recapitulated in cultured intestinal organoids [35]. The ERK pulse propagation is diminished either by an EGFR inhibitor or by a matrix metalloproteinase (MMP) inhibitor, suggesting that propagation of ERK activity pulses requires shedding of EGFR ligands and the resultant EGFR activation, as in the case of cultured epithelial cells. Since spontaneous firing of ERK activity pulses in each cell is also inhibited by an EGFR inhibitor, EGFR signaling should be a major driver of ERK activity pulses in IECs. Notably, the basal ERK activity is mediated by a different RTK, ErbB2. Therefore, ERK activity dynamics in IECs are defined by two distinct modes of ERK activity, pulse-like activity and basal activity, which are driven by EGFR and ErbB2 signaling, respectively (Figure 2) [35]. The distinct ERK activity dynamics might be attributed to different regulatory mechanisms and characteristics of the two receptors. For instance, upon ligand-induced activation, EGFR undergoes rapid internalization, while ErbB2 has no known ligands and is assumed to transmit more sustained signals [8]. In addition, ERK activation also triggers negative feedback regulation of the upstream molecules including EGFR, which should play a key role in shaping the pulsatile ERK activation pattern at different levels of the signaling cascade [6]. Importantly, ErbB2 inhibition, but not EGFR inhibition, strongly suppresses proliferation of IECs [35], suggesting that the ErbB2-dependent basal ERK activity plays a major role in promoting proliferation of IECs.

In the intestinal epithelium, constitutive activation of Wnt signaling, which is often caused by APC or β-catenin mutations, represents the initial trigger of tumorigenesis [68,69,70]. During the tumor initiation processes, ERK activity dynamics are likely to be changed to promote cell proliferation, as intestinal organoids derived from adenomas developed in Apc mutant mice [71] exhibit altered ERK activity dynamics: the ERK activity pulses are increased in an EGFR-dependent manner and the basal ERK activity is also dependent on EGFR signaling in adenoma organoids [35]. Genetic or pharmacological activation of Wnt signaling also causes similar changes in ERK activity dynamics and promotes cell proliferation in an EGFR-dependent manner. Therefore, during intestinal tumorigenesis, Wnt signaling activation alters ERK activity dynamics by augmenting EGFR signaling, which renders IECs highly dependent on EGFR signaling [35]. These findings provide a therapeutic rationale for why EGFR inhibitors can specifically target CRCs in clinical practice without causing severe damage to the normal tissues (Figure 2).

How does Wnt signaling activation augment EGFR signaling in IECs? The answers to this question lie in the transcriptional control of EGFR regulators. Wnt signaling activation upregulates two positive regulators of EGFR (Egfl6, and Troy) [72,73], and downregulates one negative EGFR regulator (Lrig3) [74] in IECs [35]. The knockdown of either Egfl6 or Troy, or exogenous expression of Lrig3 partially restores normal ERK activity dynamics in adenoma organoids [35]. In addition to the altered expression of EGFR regulators, expression of EGFR itself is also increased in adenomas compared to the normal epithelium. Therefore, altered expression of multiple EGFR regulators, together with enhanced EGFR expression, coordinately promotes EGFR-ERK signaling in adenoma cells (Figure 2) [35]. It should be noted that these EGFR regulators have been implicated in human cancers [72,75,76,77,78,79,80]. Thus, altered expression of EGFR regulators and the resultant changes in ERK activity dynamics might be prevalent in these cancers. An important remaining question regarding ERK activity dynamics in IECs is whether the pulsatile and sustained ERK activities lead to distinct outputs at the molecular level. Given that increased ERK activity pulses promote proliferation of intestinal adenoma cells, it should be important to examine whether the pulsatile nature of the ERK activity per se has some meaning in IECs.

## 5. Conclusions

The ERK pathway serves as a critical hub linking various extracellular stimuli to divergent cellular responses depending on the biological contexts. The divergent functions of the pathway are underpinned by spatiotemporal regulation of ERK activity dynamics. Fluorescent bioimaging approaches with highly sensitive ERK biosensors are the powerful and only tool to elucidate ERK activity dynamics in living cells and tissues. Emerging evidence from such bioimaging approaches suggests unexpectedly complex ERK activity dynamics and their significance in many biological processes, including cell fate decisions, cell migration, embryonic development, and tumorigenesis. Elucidation of detailed mechanisms by which ERK activity dynamics are defined and linked to specific cellular outputs would shed light on the molecular bases for many physiological and pathological processes.
